# Stone Formation from Nonabsorbable Clip Migration into the Collecting System after Robot-Assisted Partial Nephrectomy

**DOI:** 10.1155/2014/397427

**Published:** 2014-02-23

**Authors:** Ziho Lee, Christopher E. Reilly, Blake W. Moore, Jack H. Mydlo, David I. Lee, Daniel D. Eun

**Affiliations:** ^1^Department of Urology, Temple University School of Medicine, 255 S 17th Street, Suite #2101, Philadelphia, PA 19103, USA; ^2^Division of Urology, University of Pennsylvania School of Medicine, Philadelphia, PA, USA

## Abstract

We describe a case in which a Weck Hem-o-lok clip (Teleflex, Research Triangle Park, USA) migrated into the collecting system and acted as a nidus for stone formation in a patient after robot-assisted partial nephrectomy. The patient presented 2 years postoperatively with left-sided renal colic. Abdominal computed tomography scan showed a 10 millimeter renal calculus in the left middle pole. After using laser lithotripsy to fragment the overlying renal stone, a Weck Hem-o-lok clip was found to be embedded in the collecting system. A laser fiber through a flexible ureteroscope was used to successfully dislodge the clip from the renal parenchyma, and a stone basket was used to extract the clip.

## 1. Introduction

Renorrhaphy is a time-sensitive and technically challenging aspect of robot-assisted partial nephrectomy (RPN). As such, the sutures used for kidney closure are commonly secured in place with a surgical clip [1], rather than conventional knot tying. A rare postoperative complication associated with this technique is migration of the surgical clip into the urinary tract, which may cause significant morbidity for patients [2–4]. Herein, we describe a case in which a Weck Hem-o-lok clip (Teleflex, Research Triangle Park, USA) migrated into the collecting system and acted as a nidus for stone formation after RPN.

## 2. Case Report

A 52-year-old man with a history of nephrolithiasis and prostate cancer after robot-assisted radical prostatectomy presented with a small left renal mass. Abdominal computed tomography (CT) scan with and without contrast showed an enhancing 3 centimeter (cm) left middle pole renal mass that was noted to have increased in size since a prior CT scan. Subsequently, the patient underwent an uneventful left RPN. The renorrhaphy was completed in a single layer using a running 3-0 Vicryl (Ethicon, Somerville, USA) suture which was secured in place with Weck Hem-o-lok clips using sliding clip technique. Hemostasis was achieved without the use of any hemostatic agents or bolsters.

The patient's postoperative course was complicated by a perinephric hematoma that was diagnosed by CT scan. Renal angiography was negative for active bleeding, and the patient was managed with blood transfusions and close observation in the intensive care unit. Pathology indicated a 2.4 cm clear cell renal cell carcinoma, Fuhrman grade II, and negative surgical margins.

**Figure 1:**
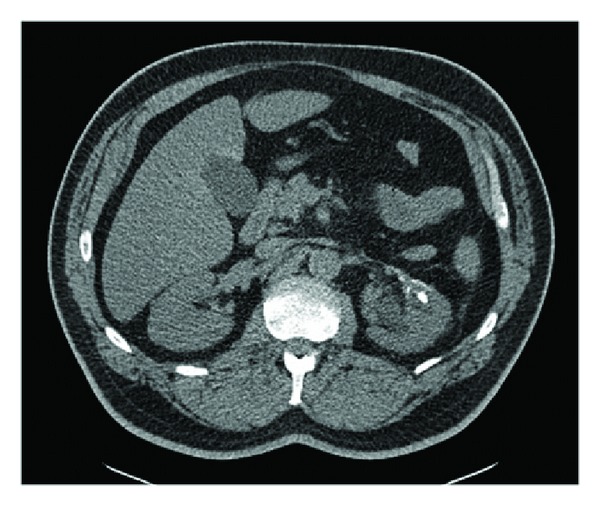
Noncontrast helical abdominal CT scan showing persistent 10 mm left renal calculus after extracorporeal shock wave lithotripsy.

**Figure 2:**
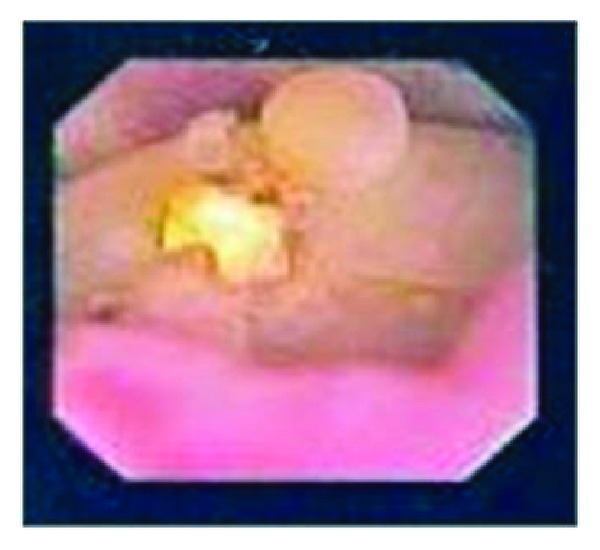
Renal calculus containing Weck Hem-o-lok clip as seen on flexible ureteroscopy.

Two years after RPN, the patient presented with left-sided colicky flank pain. Noncontrast helical abdominal CT scan showed a 6 millimeter (mm) left ureteral stone and a 10 mm left middle pole stone, associated with mild left hydroureteronephrosis. After the patient was acutely managed with left ureteral stent placement, he later underwent extracorporeal shock wave lithotripsy, which did not completely resolve his stone burden (Figure 1). The patient then underwent left flexible ureteroscopy that revealed the 10 mm renal calculus, which was adherent to the middle pole (Figure 2). Upon successful fragmentation of the stone using laser lithotripsy, a foreign body nidus consistent with a Weck Hem-o-lok clip was identified. At that time, the clip was unable to be dislodged from the collecting system. On second stage left flexible ureteroscopy, a laser fiber was used to successfully free the Weck Hem-o-lok clip from the renal parenchyma, and a basket was used to safely extract the clip. The patient was discharged the following day. Follow-up imaging showed that the patient was stone-free, and the patient noted that his flank pain had resolved.

## 3. Discussion

Surgical clips are widely used for anchoring sutures during the renorrhaphy portion of RPN because of their easy application and secure clamping. As the migration of surgical clips into the urinary tract is a rare complication after partial nephrectomy, it has only been reported in the literature in a few instances. In the first reported case, Miller et al. described a patient who experienced migration of LAPRA-TY clips (Ethicon Endo-Surgery, Cincinnati, USA) into his urinary tract after laparoscopic partial nephrectomy. The patient presented with renal colic six weeks postoperatively, and the patient spontaneously passed several remnants of the absorbable clips after two weeks of conservative management with hydration and narcotic analgesia [4]. In another case, Massoud described a patient who experienced migration of a Premium Surgiclip (Covidien, Dublin, Ireland) into his urinary tract after open partial nephrectomy. The patient presented with flank pain nine years postoperatively, and the patient spontaneously passed the titanium clip after a few days of conservative management with hydration and narcotic analgesia [3]. In the most recently reported case, Park et al. described a patient who had migration of a Weck Hem-o-lok clip into his urinary tract after laparoscopic partial nephrectomy. The patient presented with flank pain two years postoperatively, and the nonabsorbable clip was retrieved from the ureter with a stone basket [2].

Although a foreign body in the urinary tract is prone to act as a focal point for stone formation, there have been no prior reports to date of clip migration and subsequent calculus formation after partial nephrectomy. However, a few case reports have described intravesical migration of Weck Hem-o-lok clips leading to stone formation after radical prostatectomy [5, 6]. Other reports have shown that staples used during cystectomy and urinary diversions may also cause stone formation when they are in direct contact with urine [7]. Furthermore, in an investigation of various suture materials in the bladder wall of rats, Kosan et al. found that the duration of contact between foreign body and urine was the greatest predictor of stone formation [8]. Our report shows that surgical clips migrating into the urinary tract causing calculus formation after RPN may cause significant morbidity for patients.

The precise mechanism by which a surgical clip migrates into the urinary tract after partial nephrectomy is unclear. In the aforementioned case report by Miller et al., the authors conjectured that the LAPRA-TY clips may have migrated into the urinary tract through a violation of the collecting system created unknowingly by the surgeons during laparoscopic partial nephrectomy [4]. In our case, the postoperative bleed could have been indicative of an opening in our kidney closure. Also, as the renorrhaphy requires appropriate parenchymal closure pressure, another possible explanation is that the tension on the suture may have facilitated clip migration into the collecting system.

Although clip migration into the urinary tract after RPN is rare, urologists should be aware of the complication as it may cause significant morbidity for patients. One adverse potential sequela is the formation of urinary stones, which may require aggressive ureteroscopic management.
